# Liquid Metal‐Elastomer Composites with Dual‐Energy Transmission Mode for Multifunctional Miniature Untethered Magnetic Robots

**DOI:** 10.1002/advs.202203730

**Published:** 2022-09-05

**Authors:** Jiachen Zhang, Ren Hao Soon, Zihan Wei, Wenqi Hu, Metin Sitti

**Affiliations:** ^1^ Physical Intelligence Department Max Planck Institute for Intelligent Systems 70569 Stuttgart Germany; ^2^ Department of Biomedical Engineering City University of Hong Kong Hong Kong SAR China; ^3^ Institute for Biomedical Engineering ETH Zürich Zürich 8092 Switzerland; ^4^ School of Medicine and College of Engineering Koç University Istanbul 34450 Turkey

**Keywords:** liquid metal, magnetic soft composite, miniature mobile robotics, soft robotics, wireless energy transmission

## Abstract

Miniature untethered robots attract growing interest as they have become more functional and applicable to disruptive biomedical applications recently. Particularly, the soft ones among them exhibit unique merits of compliance, versatility, and agility. With scarce onboard space, these devices mostly harvest energy from environment or physical fields, such as magnetic and acoustic fields and patterned lights. In most cases, one device only utilizes one energy transmission mode (ETM) in powering its activities to achieve programmed tasks, such as locomotion and object manipulation. But real‐world tasks demand multifunctional devices that require more energy in various forms. This work reports a liquid metal‐elastomer composite with dual‐ETM using one magnetic field for miniature untethered multifunctional robots. The first ETM uses the low‐frequency (<100 Hz) field component to induce shape‐morphing, while the second ETM employs energy transmitted via radio‐frequency (20 kHz–300 GHz) induction to power onboard electronics and generate excess heat, enabling new capabilities. These new functions do not disturb the shape‐morphing actuated using the first ETM. The reported material enables the integration of electric and thermal functionalities into soft miniature robots, offering a wealth of inspirations for multifunctional miniature robots that leverage developments in electronics to exhibit usefulness beyond self‐locomotion.

## Graphical abstract

This work presents a dual‐energy transfer pipeline (dual‐ETP) soft material by embedding liquid metal into magnetically responsive elastomer. This material integrates dual‐ETP using global magnetic fields and does not complicate the setup. While maintaining all the versatile advantages of conventional magnetically responsive soft materials, this material obtains additional energy in electricity to power onboard electronics and heat for hyperthermia treatment.

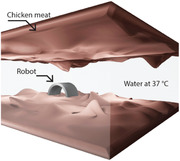

## 1 Introduction

Remotely actuated miniature untethered devices are promising in a wide range of potential applications.^[^ ^1^, ^2^ ^]^ In particular, these miniaturized devices could bring radical improvements to biomedical functions and play a pivotal role in modern medical operations, e.g., minimally invasive diagnostics^[^ ^3^, ^4^ ^]^ and therapeutics.^[^ ^5^, ^6^, ^7^ ^]^ With a body size ranging from a few millimeters down to several micrometers, these devices have extremely limited onboard space, and thus most of them do not carry any onboard power source. Instead, they harvest energy via a wide diversity of mechanisms: from environmental sources such as temperature changes,^[^ ^8^ ^]^ chemical reactions,^[^ ^9^, ^10^ ^]^ mechanical vibrations,^[^ ^11^ ^]^ and living microorganisms,^[^ ^12^, ^13^ ^]^ or from externally applied physical fields, such as magnetic,^[^ ^14^ ^]^ acoustic,^[^ ^15^ ^]^ and optical fields.^[^ ^16^, ^17^, ^18^ ^]^ We refer to such a path from the energy source to energy consumption for functionalities as an energy transmission mode (ETM). The representative cases are summarized in Table S1 (Supporting Information), which shows that, conventionally, each ETM only accommodates one or a few specific functionalities.

Magnetic field stands out from others because of its unique advantages of being easy to generate and control using electromagnetic coils or permanent magnets, being able to penetrate many substances and exerting high‐speed and simultaneous forces and torques, and being relatively safe for the human body.^[^ ^19^ ^]^ Magnetic small‐scale devices rely on magnetically responsive smart materials that possess a monotonous and deterministic magnetic‐responsiveness, which serves as the backbone of their working principles. As a result, single device has limited functionalities and capabilities, and has little or no tunability and reconfigurability to offer without radical changes. Further advancement in this emerging field will appreciate an enriched and diversified functionality catalog with various functional units. But it is nontrivial to power a device integrating different units that are driven by diverse energy sources via distinct working principles. In particular, while these magnetically controlled miniature robots demonstrate multimodal locomotion capabilities, many tasks still demand electrical functional components that require onboard electrical currents. A brutal approach to activate the components relying on different ETMs is superimposing multiple setups onto the same workspace. Using this approach, additional secondary stimuli such as heat,^[^ ^20^, ^21^ ^]^ light,^[^ ^22^ ^]^ and chemical reactions^[^ ^23^ ^]^ have been integrated to magnetic miniature robots. However, this implementation complicates the system and limits the applicability of such materials. Considering the use of magnetic fields is predominantly motivated by its safe penetration into biological tissues, adding heat, light, and chemical signals to the system defeats the purpose. A more preferable solution should obtain energy from a single source for different functionalities.

Outside the field of magnetic miniature robotics, smart materials researchers have embarked on a quest to integrate multiple functional materials together for unrivalled capability and versatility, utilizing the respective advantages of each material and promoting concerted development of smart composite materials for sophisticated, realistic tasks in unstructured environments.^[^ ^24^, ^25^ ^]^ In this process, liquid metal (LM) has gained wide popularity due to its unique advantages as a compliant and flexible material with metallic properties, such as its exceptional electric and thermal conductivity,^[^ ^26^ ^]^ self‐healing properties,^[^ ^27^ ^]^ and self‐sensing actuation capability.^[^ ^28^ ^]^ And LM has also been integrated with magnetorheological elastomers for exotic material properties, such as positive piezo‐conductivity.^[^ ^29^ ^]^

This work introduces an LM‐elastomer monolithic composite that obtains energy from a single externally applied magnetic field and converts it to electricity for functionalities such as powering onboard electronic units or performing local heating, while maintaining the versatile locomotion and magnetically controlled shape‐morphing capabilities of the material. The material is activated and controlled by an externally applied global magnetic field ${\vec{B}}_{g}$, which is a superposition of two constituent magnetic fields, i.e., ${\vec{B}}_{g}={\vec{B}}_{\mathrm{low}}\;+{\vec{B}}_{\mathrm{rf}}$. One field component has a low frequency (<100 Hz) and is denoted as ${\vec{B}}_{\mathrm{low}}$, while the other one has a high changing rate within the range of radio frequency (RF, 20 kHz–300 GHz) and is thus denoted as ${\vec{B}}_{\mathrm{rf}}$. ${\vec{B}}_{\mathrm{low}}$ induces shape‐morphing behaviors of the material for locomotion, which has been well‐studied and widely employed in miniature robotics.^[^ ^1^, ^14^, ^30^ ^]^

The reported material picks up additional energy from ${\vec{B}}_{\mathrm{rf}}$ and converts it to different forms other than mechanical energy. The additional energy in the form of electricity can be used to enable a diverse set of electronic units. Moreover, this energy could be further converted into other forms, such as heat via Joule heating and light via light‐emitting diode (LED) or laser diode. The energy in the form of heat was employed to enable heat treatment in a phantom emulating biomedical application scenarios. The additional ETM exhibited by the reported material could benefit future multifunctional robots bearing distinctive functional units. The versatility in the energy forms could enable various functional units as well as power other functional devices in the vicinity.

## 2 Design of the LM‐Elastomer Composite

**Figure 1:**
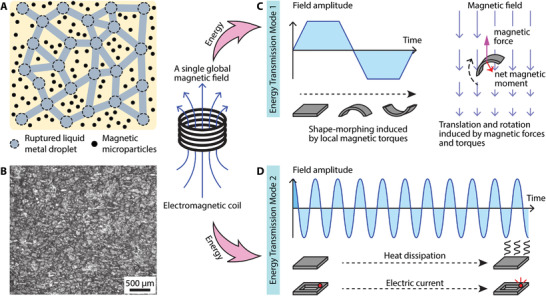
Schematics illustrating the design and two working principles of the reported soft composite material. A) Schematic illustration of the reported magnetic liquid metal‐elastomer composite. B) An optical top‐view microscope image of the composite material. C) The first energy transmission mode is established by a low‐frequency component of the magnetic field to deliver mechanical energy for shape‐morphing. D) The newly enabled second energy transmission mode is established by an RF fast‐changing component of the magnetic field to deliver heat and electric current for hyperthermia and onboard electronics.

The reported composite consists of an elastomer matrix with embedded hard magnetic microparticles (MMPs) and LM droplets, both of which are homogeneously distributed within the material. Schematics of the material concept and its optical microscopy are shown in **Figure** **1**A,B, revealing the homogeneous distribution of LM droplets at a size magnitude of hundreds of microns. Polydimethylsiloxane (PDMS) was chosen as the base elastomer due to its advantages in low modulus, high strain limit, and biocompatibility. Neodymium‐iron‐boron (NdFeB) MMPs were employed for their strong hard‐magnetic properties and commercial availability. Eutectic Gallium‐Indium (EGaIn) was utilized as the LM due to its low melting point, high electric conductivity, and low viscosity.

NdFeB microparticles have an average diameter of 5 µm. These embedded MMPs bear a pre‐programmed magnetization profile $\vec{m}$ (magnetic moment) across the material, making the overall soft composite material shape‐programmable. In response to ${\vec{B}}_{\mathrm{low}}$, the dispersed MMPs experience distributed torques $\vec{\tau }$ and forces $\vec{F}$ determined by equations of $\vec{\tau }=\vec{m}\;\times {\vec{B}}_{\mathrm{low}}$ and $\vec{F}=\;\nabla \left(\vec{m}\cdot {\vec{B}}_{\mathrm{low}}\right)$, respectively. These forces and torques are then transferred to the supporting elastomer matrix, inducing shape‐morphing behaviors of the device and consequent functionalities, such as locomotion (Figure 1C).

**Figure 2:**
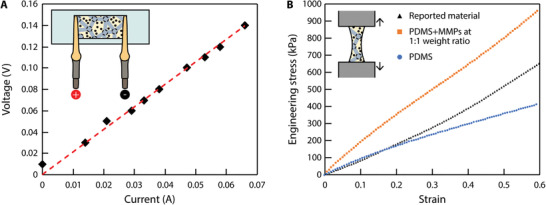
Electrical and mechanical characterization of the soft composite material. A) Measurement of the resistivity of the reported material (35  ×  18  ×  0.22 mm^3^). Each data point represents the average of three measured values. The variation is smaller than the marker size. B) Measurement of Young's moduli of the reported material and two other reference materials, i.e., PDMS (10:1 mass ratio between base and curing agents) and PDMS mixed with NdFeB MMPs at 1:1 weight ratio.

On the other hand, the ruptured LM droplets^[^ ^27^ ^]^ form a conductive network within the material, establishing a second ETM to pick up energy from ${\vec{B}}_{\mathrm{rf}}$. The current–voltage curve of a sheet‐shaped proposed material of 35  ×  18  ×  0.22 mm^3^ is presented in **Figure** **2**A. The results suggest a resistance of 2.1 Ω and a resistivity of 2.4  ×  10^–4^ Ω m. This second ETM provides electricity, which can be employed to power onboard electronics such as LED. It also can be further converted into heat via Joule heating for possible treatment functions, such as hyperthermia and thermal ablation.

The mechanical properties of the reported material were characterized by stretching up to a strain of 0.6. The experimentally measured stress–strain curve was reported in Figure 2B, together with the curves of PDMS and a composite material of PDMS and MMPs at a 1:1 mass ratio as references. The results indicate that the presence of LM droplets within the material counteracts the stiffening effects caused by the introduction of MMPs, giving the composite material a similar Young's modulus with the reference PDMS material up to a strain of about 0.2. Further increasing the strain up to 0.6, the reported material maintains a relatively linear stress–strain curve and a consistent Young's modulus. However, the stress–strain curve of the reference PDMS material “flattens out,” suggesting a reduced Young's modulus. Therefore, the versatile and agile locomotion of the existing magnetoelastomer is well retained.

## 3 Multifunctional Robot Demonstrations

**Figure 3:**
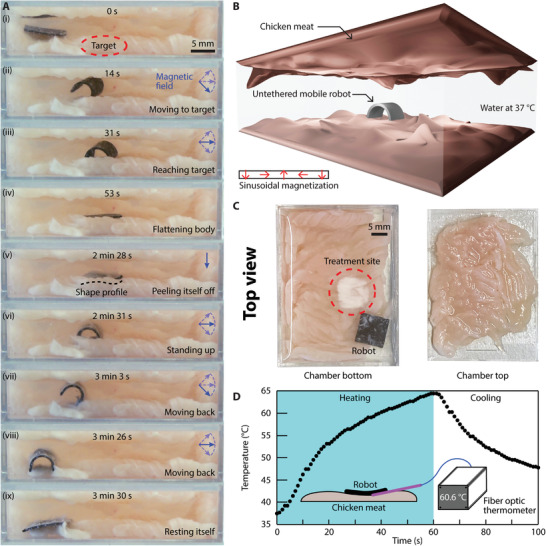
A sheet‐shaped, untethered miniature magnetic soft robot performing heat treatment on chicken breast meat in water. A) Video snapshots of the robot walking inside the tissue‐covered chamber (see Video S1, Supporting Information). B) 3D schematic illustrating the experimental setup. The untethered robot moved inside a chamber filled with distilled water preheated to 37 °C with walls made of chicken meat. The inset shows the side‐view sinusoidal magnetization profile of the robot. C) Top‐view images of the bottom and top surfaces of the chamber after the experiment. D) Experimentally measured temperature profile of the treatment site during the experiment in response to a ${\vec{B}}_{\mathrm{rf}}$ with a strength of 86.2 kA m^–1^ alternating at a frequency of 337 kHz.

As the reported material enables an additional ETM from ${\vec{B}}_{\mathrm{rf}}$, we demonstrate the consequent advantages by creating two proof‐of‐concept multifunctional robots. While maintaining the conventional versatile mobility shown before on magnetic soft robot with one ETM,^[^ ^30^, ^31^, ^32^ ^]^ these two robots take advantage of the newly available ETM for additional functionalities of hyperthermia and powering onboard electronics, respectively. As shown in **Figure** **3**, a square sheet robot made of the reported material was prototyped with geometric dimensions of 9  ×  9  ×  0.5 mm^3^. The robot bears a sinusoidal magnetization profile along one of its dimensions to enable shape‐morphing for locomotion under low‐frequency magnetic fields, as shown in Figure 3B.^[^ ^33^ ^]^ The robot worked inside an enclosed workspace filled with distilled water at 37 °C to mimic hard‐to‐reach cavities inside the human body. The fresh chicken breast meat was placed at the floor and also the ceiling of the cavity.

First, the robot was wirelessly controlled by an externally applied ${\vec{B}}_{\mathrm{low}}$ to move within the chamber, reach a designated location, and rest upon a targeted tissue site (Figure 3Ai–iv). Then, ${\vec{B}}_{\mathrm{rf}}$ was applied for 60 s to induce eddy currents onboard the robot that generated substantial heat (Figure 3Aiv,v). The targeted tissue site within the immediate vicinity of the robot was heated up through contact conduction. The resultant temperature rise was sufficient enough to induce protein denaturation within the chicken breast meat at the targeted site, mimicking localized hyperthermia and thermal ablation treatment^[^ ^34^ ^]^ in clinical settings (Figure 3C). Figure 3C also shows untreated chicken breast meat as the contrast. The temperature profile during the hyperthermia treatment was further captured by a fiber optic thermometer (FOTEMP‐PLUS, Fiber Optic Thermometer, Micronor Sensors). The representative temporal temperature profile was displayed in Figure 3D, showcasing a rapid temperature rise at the meat surface enabled by the robot. Note that the temperature was not controlled in real‐time, instead the temperature was measured by the fiber optical thermometer and the data was analyzed afterward. The fiber optic thermometer was employed because it was not affected by ${\vec{B}}_{\mathrm{rf}}$ (see Section S2, Supporting Information). This temperature profile can be tuned by adjusting the power setting of the ${\vec{B}}_{\mathrm{rf}}$ generator and the robot's exposure time to ${\vec{B}}_{\mathrm{rf}}$.

After the heat treatment, the robot stuck to the meat due to the heating. But thanks to its sinusoidal magnetization profile, the robot then deformed in response to ${\vec{B}}_{\mathrm{low}}$. Starting from its corner, the robot gradually peeled itself off the meat, as shown in Figure 3Av. Next, the robot moved back to its starting position under the wireless maneuvering of ${\vec{B}}_{\mathrm{low}}$ (Figure 3Avi‐ix). This demonstrated proof‐of‐concept heat treatment conducted by the exemplar robot showcased the potential of the reported material in hyperthermia and thermal ablation, which are transformative procedures for disease treatment in modern healthcare.^[^ ^34^ ^]^ Such a procedure holds a promise for treating cancers such as prostate cancer.^[^ ^35^, ^36^ ^]^ Even though the robot was submerged in water, a rise from 37 °C to about 65 °C was still experimentally achieved and it far exceeds the required temperature for hyperthermia, which is between 40 and 43 °C.^[^ ^37^ ^]^ In the future, the heat could also be utilized as a local heat source to activate self‐healing materials^[^ ^38^ ^]^ or shape memory alloy (SMA). See Section S3 (Supporting Information) for a preliminary investigation of employing the reported material as a localized heat source to activate liquid crystal elastomer (LCE) sheets for cargo lifting.

**Figure 4:**
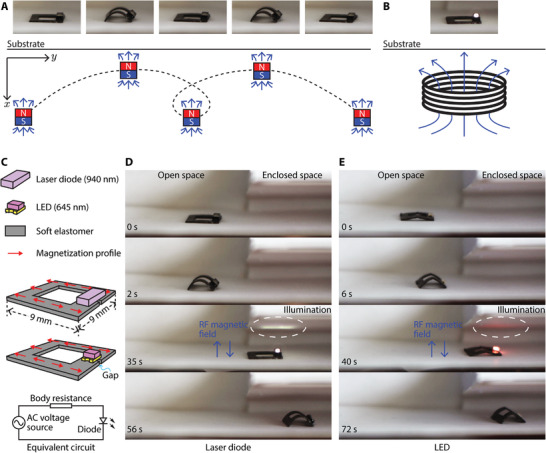
Untethered miniature soft robot surface‐crawling into an enclosed space using magnetic field‐based actuation and powering its onboard electronics using RF power transfer. A) The soft robot deformed its body for surface‐crawling locomotion using an external low‐frequency magnetic field waveform. B) The robot generated onboard electric current for powering an LED via the externally exerted RF fast‐changing magnetic field. C) Schematics illustrating two robot designs and their equivalent circuit. Two robots were fabricated, with the only difference being the onboard electronic units. A series of experimental snapshots are presented to show a robot moved into an enclosed space and powered a D) laser diode and E) an LED. The ${\vec{B}}_{\mathrm{rf}}$ has a frequency of 337 kHz. It has a strength of 46.8 and 24.3 kA m^–1^ for the laser diode and the LED experiments, respectively. Videos of (D) and (E) are available in Videos S2 and S3 (Supporting Information), respectively.

As shown in **Figure** **4**, a second exemplar robot was prototyped to power onboard electronics. It has geometric dimensions of 9  ×  9  ×  0.4 mm^3^. The ring‐shaped robot has a broken gap beneath the onboard electronic unit. This gap forces the induced electric currents inside the robot body to go through the electronic unit and thus power it, which is a LED (OSRAM CHIPLED 0805) or a laser diode (OSRAM PLPVQ 940A). Similar to the first exemplar robot, this ring‐shaped robot also bears a programmed magnetization profile that enables it to respond to the externally applied ${\vec{B}}_{\mathrm{low}}$ via shape‐morphing for locomotion, as shown in Figure 4A.

The robot locomoted on a flat substrate in response to ${\vec{B}}_{\mathrm{low}}$, which was applied by moving a permanent magnet beneath the substrate in “O” shapes. The robot moved like an inchworm by repeatedly shrinking and expanding its body while alternating the anchoring feet.^[^ ^1^ ^]^ After the robot reached its target location, ${\vec{B}}_{\mathrm{rf}}$ was applied to activate the second ETM of the robot and power the onboard electronics, see Figure 4B. In the experiments, two robots were fabricated from the same design with the only difference of being the first one carrying a laser diode (PLPVQ 940A, OSRAM Opto Semiconductors) while the second one carrying an LED (CHIPLED 0805, OSRAM Opto Semiconductors) as the exemplar onboard functional components. The effective electric circuit of the robots is presented in Figure 4C. In the two demonstrations with a laser diode and an LED, the robots were wirelessly controlled to move from an open space into an enclosed space. The laser diode and LED were then activated to shine laser and visible red light to illuminate the enclosed workspace. Afterward, the robots continued to move further into the enclosed space. The process of this experiment was shown in Figure 4D,E. Experimental videos of this experiment with an onboard laser diode and LED are available in Videos S2 and S3 (Supporting Information), respectively.

Although the laser diode and LED are similar electronic units, the laser diode requires a significantly higher voltage to activate and consumes much more energy. This result suggests that the reported untethered robots are able to generate onboard electric currents to power onboard electronics, even the ones that demand relatively high power. Besides employing the laser or visible light for direct functionalities, the laser diode and LED units could also further extend the second ETM to convert the received magnetic fieldenergy into light energy and then actuate or trigger other functional components onboard or in the vicinity of the robot. Note that the laser diode and LED are special in terms of their electric characteristics of being compatible with alternating electric currents. Other electronic units will need an additional rectifying circuit or unit being integrated into the robot to function properly. Enabling onboard electronics for shape‐morhping miniature robots lays the foundation for a variety of functionalities, such as localized sensing, wireless communications, illumination, etc., thanks to the diverse capabilities of modern electronic components.

It is noteworthy that the different geometry designs of the exemplar robots in the aforementioned experiments decided which direction the obtained energy via the second ETM flowed toward. More specifically, the first robot has a fully filled square body, which facilitated the formation of eddy currents to convert electric energy to heat. On the contrary, the second robot design has a ring‐shaped body with a broken gap. Its body obtained the same amount of energy as the first robot, but instead of dissipating the energy via heat, its body forced the induced electric currents to flow around its ring‐shaped body and pass through the onboard electronic units mounted atop the broken gap. Electronic units mounted on the body of the first robot would not work since the units were short‐circuited, and energy was dissipated as heat. Future study could further exploit the design freedom in geometry to enable different electronic applications.^[^ ^27^ ^]^ Superposing two magnetic fields of distinct frequencies has been reported previously.^[^ ^39^, ^40^ ^]^ However, previous studies only employed a fast‐changing magnetic field for the purpose of heating up Fe_3_O_4_ nanoparticles to soften the base material of shape memory polymer or to induce bilayer deformation of hydrogel, without further exploiting the wireless ETM for additional functionalities.

## 4 Conclusion

This work reports an LM‐elastomer composite for multifunctional miniature untethered magnetic robots. The material has a soft polymer matrix with uniformly distributed and mechanically sintered LM droplets in it. In contrast to previous approaches using embedded wires^[^ ^41^ ^]^ or rigid filler particles^[^ ^42^ ^]^ for conductivity, the embedded LM in the reported material forms an internal conductive network while preserving the homogeneous bulk properties, material softness, and the magnetic responsiveness. This monolithic material obtains energy from an externally applied global magnetic field via two ETMs and converts energy into elastic, mechanical, electrical, and thermal energy forms, which are subsequently utilized to achieve functionalities such as interacting with the environment, powering onboard electronics, and applying local heat treatment. Although a previous study^[^ ^29^ ^]^ has reported the use of magnetic particles and LM in a polymer matrix, it did not exploit the controllable versatile shape‐morphing for active functionalities or wireless energy transmission, but only focused on the passive sensing capability on tethered devices. Beyond the demonstrated cases, the material's magnetization can also be programmed to arbitrary profiles to enable other shape‐morphing behaviors suitable for various tasks.^[^ ^30^ ^]^

This work develops a smart material to enrich and diversify the energy forms available for miniature untethered robots, extending the design space and enabling robots with high complexity and multifunctionalities. Proof‐of‐concept mobile robots were experimentally demonstrated for potential soft robotic and biomedical applications, validating the feasibility of the proposed material in employing energy in different forms. Further development of each and every demonstrated robot warrants its own follow‐up study. Beyond the multifunctionalities shown on individual robot, following research will foster a scheme that employs the reported material to create energy‐relaying agents to power other stimuli‐responsive materials or other miniature robots that demand energy in different forms. See Section S3 (Supporting Information) for a preliminary case study.

## 5 Experimental Section

### Materials

EGaIn liquid metal and NdFeB MMPs were purchased from Merck KGaA and Magnequench International, LLC, respectively. MMPs and eGaIn were mixed together with the base and curing agent of PDMS (Sylgard 184, Dow Corning). The weight ratio between the different components of the composite was set to be 11:110:10:1 (MMPs : LM : PDMS base agent : PDMS curing agent), corresponding to a volume ratio of 5%, 59%, and 36% (MMPs : LM : PDMS) based on the density value (7.61 g cm^−3^ for MMPs, 6.25 g mL^−1^ for LM, and 1.03 g cm^−3^ for PDMS) provided by their respective manufacturers. MMPs were first thoroughly mixed with PDMS before eGaIn was added. As MMPs were not magnetized at this stage, they did not form chains and a homogeneous mixture could be easily obtained. Since the volume of eGaIn used in fabrication was very small, the mixture was manually stirred until an emulsion was formed and droplets of eGaIn were no longer visible. The LM formed droplets at the scale of 100 µm as shown in Figure 1B. The mixture was then degassed for 30 min to remove trapped air and poured into a mold. Excess material was removed using a razor blade via scraping. The mold with mixture in it was plated on a hot plate set at 90 °C for 2 h to cure the PDMS.

### Robot Fabrication

The cured film of the reported material was laser‐cut into desired shapes to make the exemplar robots. For this work, the heat‐generating robot has a dimension of 9  ×  9  ×  0.5 mm^3^ and the two electricity‐generating robots have a dimension of 9  ×  9  ×  0.4 mm^3^. The parts being cut from the film was dipped into a mixture of the base and curing agents of PDMS (weight ratio of 10:1) to seal the openings at the exposed cross‐sectional surface due to laser‐cutting, preventing the leakage of LM during experiments. Excess material was removed to ensure that this step adds no additional thickness to the sample film. Before laser‐cutting, the piece was uniformly compressed using a standard weight of 1 kg with an acrylic cube of 1 cm^2^ surface area in between, generating about 100 kPa to rupture the initially insulating oxide skin of the LM droplets and form a conductive network within the material. As the rupture happened immediately after the pressure was applied, no additional wait time was added. The acrylic cube was placed to the next region to continue this process until the whole film had been covered. This process is known as “mechanical sintering” and has been proposed and utilized in previous studies.^[^ ^27^, ^43^ ^]^ Characterization results in a preceding study indicate good reproducibility of the material's electrical property using this sintering strategy.^[^ ^27^ ^]^ The material was deformed to a certain shape and placed inside a vibrating sample magnetometer (VSM, EZ7, Microsense), which generates a strong magnetic field (1.8 T) to program the desired magnetization profile onboard. More details of this magnetizing process are available in the previous work.^[^ ^1^ ^]^ The robots that carry onboard electronics were fabricated by attaching the corresponding electronic components to the robots and employing uncured PDMS mixture as the bonding agent following the procedure described in the previous work.^[^ ^30^ ^]^

### Magnetic Field Generation

The quasi‐static magnetic field ${\vec{B}}_{\mathrm{low}}$ was generated by a permanent handheld magnet (Neodymium, nickel‐plated, Supermagnete). The RF fast‐changing magnetic field ${\vec{B}}_{\mathrm{rf}}$ was generated by an induction heater with controllable power output and period (EASYHEAT induction heating system, Ambrell, Ltd.).

## Conflict of Interest

The authors declare no conflict of interest.

## Supporting information

Supporting Information

Supplemental Video 1

Supplemental Video 2

Supplemental Video 3

## Data Availability

The data that support the findings of this study are available from the corresponding author upon reasonable request.
