# Supplementary material for: Liquid Metal‐Elastomer Composites with Dual‐Energy Transmission Mode for Multifunctional Miniature Untethered Magnetic Robots
Source: Adv Sci (Weinh). 2022 Sep 5;9(31):2203730. doi: 10.1002/advs.202203730 (PMC9631051; doi:10.1002/advs.202203730)
Supplement: Supplementary file 1 [file ADVS-9-2203730-s004.pdf]

Supplementary Information

Liquid metal-elastomer composites with dual-energy transmission mode for multifunctional miniature untethered magnetic robots

1. Theoretical analysis and experimental calibration of the second energy transmission mode (ETM)

Ruptured LM droplets within the reported material form a conductive network inside the material. When being exposed to an RF field component $\vec{B}_{\mathrm{rf}}$, electric currents emerge within this network, enabling the second energy transmission mode (ETM). In theory, the RF field component $\vec{B}_{\mathrm{rf}}$ leads to a rapid change of the magnetic flux $\emptyset$ through the area enclosed by the outer contour of the robot. Based on Faraday's law of induction, the induction voltage ε equals to the rate of change of the magnetic flux, as described in the following equation:

$$\varepsilon = \frac{\Delta\emptyset}{\Delta t}$$

This ETM is experimentally generated by a commercially available induction heater (details available in **Experimental Section** – Magnetic field generation). The induction heater outputs an alternating magnetic field at a fixed frequency, which is decided by the properties of the specific probe attached to it. In this study, the probe used is made of a hollow copper wire with 0.9 mm diameter. The wire was bent into 3 turns with an outer diameter of 42 mm and an inner diameter of 32 mm, see **Figure S1**A. The fixed frequency of the RF magnetic field $\vec{B}_{\mathrm{rf}}$ is 337 kHz.

The induction heater only offers one control parameter I_{setting} to control the output level. Note that this I_{setting} is not the current running inside the probe coil. Instead, it is just a numerical

setting to control the power of the heater. The current running inside the probe coil is denoted as I_{probe}. A calibration experiment was conducted to relate the current setting I_{setting} to the resultant strength of $\vec{B}_{\text{rf}}$. A single-turn copper wire coil was placed coaxially with the probe coil as a receiver coil, which was then connected to an oscilloscope for signal observation and recording, see **Figure S1**B. The receiver coil has an area of 159.3 mm^2. The separation distance between the receiver coil and the top surface of the probe coil is 3 cm. Assume the current running inside the probe coil is described as $I_{\text{probe}} = I_0 \sin(2\pi f t)$, where I_0 is the current amplitude, f is the frequency, and t is the time variable. The magnetic flux density of $\vec{B}_{\text{rf}}$ at a distance z along the central axis of the probe coil generated by the current I_{probe} is calculated based on a simplified case of the Biot-Savart law:

$$B_z = \frac{\mu_0}{2} \cdot \frac{R^2}{(z^2 + R^2)^{1.5}} \cdot I_{\text{probe}} = K \cdot I_{\text{probe}}$$

where $\mu_0 = 4\pi \times 10^{-7}$ H/m and $R = 18.5$ mm is the radius of the probe coil. $K = \frac{\mu_0}{2} \cdot \frac{R^2}{(z^2+R^2)^{1.5}}$ is a constant. Thus, it can be written in a simplified form as $B_z = B_0 \sin(2\pi f t)$, where $B_0 = K \cdot I_0$. Using Faraday's law of induction, the induction voltage ε picked up by the receiver coil is calculated as

$$\varepsilon = \frac{\Delta\emptyset}{\Delta t} = S \cdot \frac{\mathrm{d}B_z}{\mathrm{d}t} = 2\pi f \cdot S \cdot B_0 \cos(2\pi f t) = 2\pi f \cdot S \cdot K \cdot I_0 \cos(2\pi f t)$$

where S is the area of the probe coil.

For calibration purposes, the induction heater was set to a specific value, e.g., I_{setting} =749.7 A. Once the heater was turned on, the receiver coil picked up a signal due to the magnetic induction, and the oscilloscope displayed a sinusoidal signal on the screen. We measured the peak value of the induction voltage ε, e.g., 6.64 V. Using aforementioned equations, the corresponding current I_0 running inside the probe coil was calculated, e.g., $I_0 = 2.29$ kA.

Then, the strength of $\vec{B}_{\mathrm{rf}}$ can be calculated using the aforementioned equation by substituting the specific values of z and I_0. For all experiments, we measured the peak value of the sinusoidal signal displayed by the oscilloscope, and we calculated the corresponding I_0, then eventually we obtained the strength of $\vec{B}_{\mathrm{rf}}$ applied to the robot.

2. Experimental characterization of the temperature rise during heat treatment

The first exemplar robot conducted heat treatment at a designated target location of a sealed meat chamber filled with distilled water. The meat chamber was made of fresh chicken breast meat. And the water was preheated to 37 °C prior to the experiment. As illustrated in the inset of **Figure 3**C, a fiber optic thermometer measured the temperature rise at the surface of the target location during experiments. Besides the temperature profile presented in **Figure 3**C, repeated experiments were conducted with the same setup. The new fresh chicken breast meat was replaced each time to ensure the same initial experimental conditions. The temperature profiles of additional tests are reported in **Figure S2**.

3. Investigation of the possibility of employing the reported material as a power relay

The reported material is capable of obtaining energy via the additional ETM activated by $\vec{B}_{\mathrm{rf}}$. The harvested energy is in the form of an electric current, which can be further converted to heat via Joule heating or light via LED or laser diode. Thus, it opens up the possibility of employing the reported material as a power relay to activate other stimuli-responsive materials for various functionalities. This section demonstrates a proof-of-concept experiment using liquid crystal elastomer (LCE) strips placed atop the reported material. The LCE, upon activation by heat, deforms and lifts a ceiling panel made of polystyrene (PS).

The LCE strips were made of the LCE material reported in our previous study[24], and it has a classic splay director alignment across its thickness. When being heated up, the alignment is disrupted, and one surface contracts while the opposite expands, causing the strip to bend. Four LCE strips were placed atop a square robot made of the reported material with a dimension of $9 \times 9 \times 0.5$ mm^3. The robot bears the same sinusoidal magnetization profile as the one shown in **Figure 3**A. A permanent magnet generated $\vec{B}_{\mathrm{low}}$ to deform the robot and thus move it on a flat substrate from an open space into an enclosed space with a hanging ceiling. Then, the robot was activated by applying $\vec{B}_{\mathrm{rf}}$ (546 A, 337 kHz, duration of 17 seconds). The robot heated up and thus activated the LCE strips carried onboard. The LCE strips bent upwards and lifted the ceiling, until the ceiling fell off the edge of the supporting wall. This process is illustrated by side-view images captured during this experiment, as shown in **Figure S3**.

Table S1. A non-exhaustive summary of the energy transmission mode (ETM) of some exemplar miniature robots.

Robot	Energy transmission mode (ETM)	Functionalities	Potential applications	References
Millimeter-scale shape-morphing robots	Using the energy obtained from an externally applied ***magnetic field*** to induce body deformation, during which process forces and torques are being exerted to the environment.	Locomotion and object manipulation	Biomedical applications such as targeted drug delivery	[1, 14]
Self-propelled protein motors	Utilizing the energy obtained from ***chemical reactions*** to create a surface tension gradient and further generates Marangoni forces for propulsion.	Locomotion	Environmental remediation, microrobot powering, and cargo delivery applications	[10]
Somatosensory light-driven robots (SLiRs)	Applying the energy obtained from ***patterned light*** via photo-thermomechanical conversion to mechanical bending, activating shape-morphing of various robots.	Locomotion and object manipulation	Active human-robot interaction, wearable robot, environmental data collection robot, and closed-loop control of actuation and sensing system	[18]
Starfish larva-inspired synthetic ciliary bands	Employing the energy ontained from an ***ultrasound field*** to drive bulk fluid motion via acoustically actuated small-amplitude oscillations of synthetic cilia.	Locomotion and microparticle trapping	Microfluidics and biomedical applications	[15]
Hybrid sperm micromotor	Spending the energy obtained from the beating of a ***sperm flagellum*** to propel the device to swim against flowing blood	Locomotion and cargo delivery	Treatment of blood clots or other diseases in the circulatory system	[13]

Note: Magnetic steering is not counted into the discussion of ETMs because it does not directly power a robot’s activities.

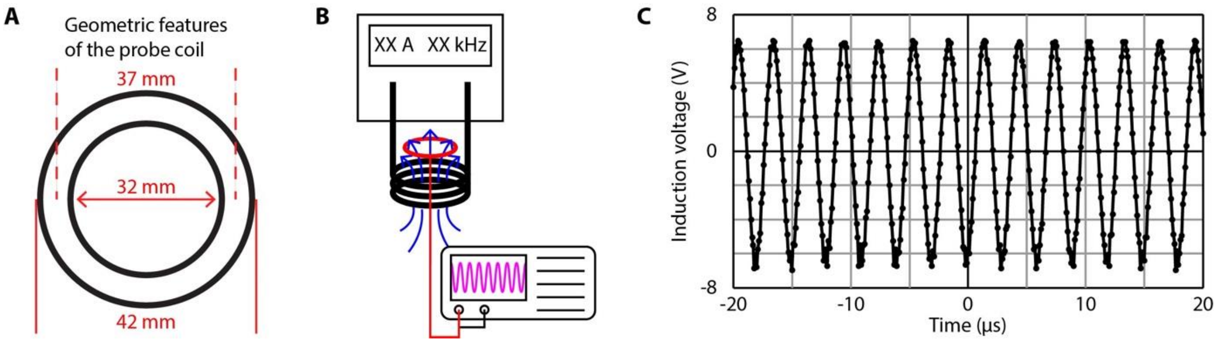

Figure S1. Setup of the calibration experiment for the RF magnetic field. A) The geometric dimensions of the probe coil of the induction heater. **B)** Schematic illustrating the setup of the induction heater and the custom-made receiver terminal connected to an oscilloscope. **C)** A representative profile curve of the picked-up induction voltage by the receiver terminal and displayed on the oscilloscope screen at a maximum power output of the induction heater.

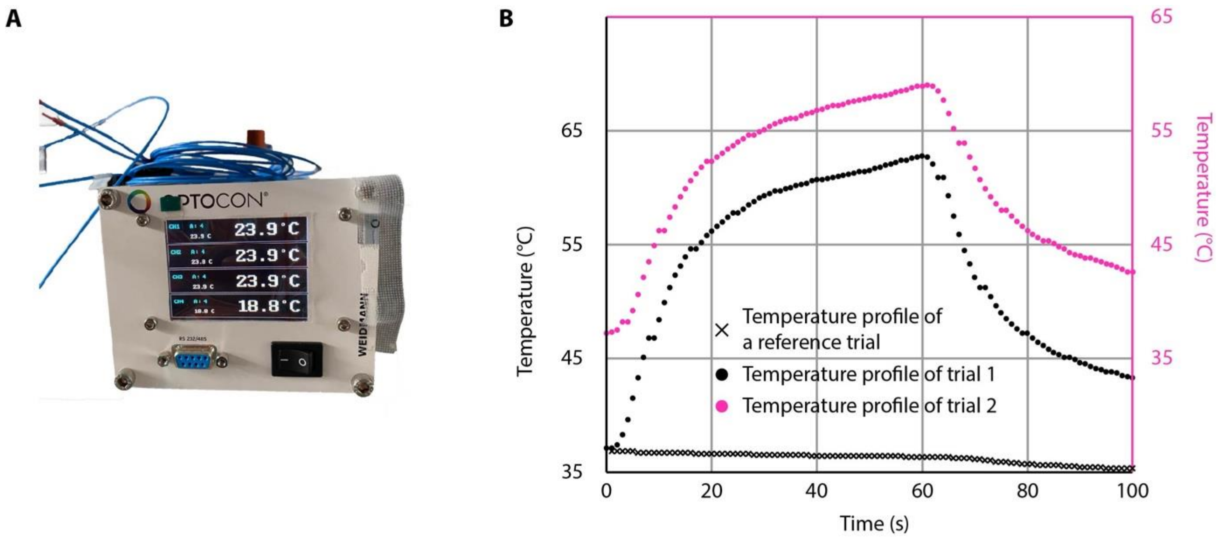

Figure S2. Fiber optical thermometer and measured temperature profiles. A) A photograph of the employed fiber optical thermometer in the reported experiments. This thermometer is capable of measuring four channels simultaneously and outputting the recording to a computer via RS232/485 interface. **B)** Experimentally measured temperature profiles of one reference trail without applying $\vec{B}_{\mathrm{rf}}$ and two additional trials besides the one reported in **Figure 3**C. The temperature profile of trial 2 is shifted upwards for better visibility.

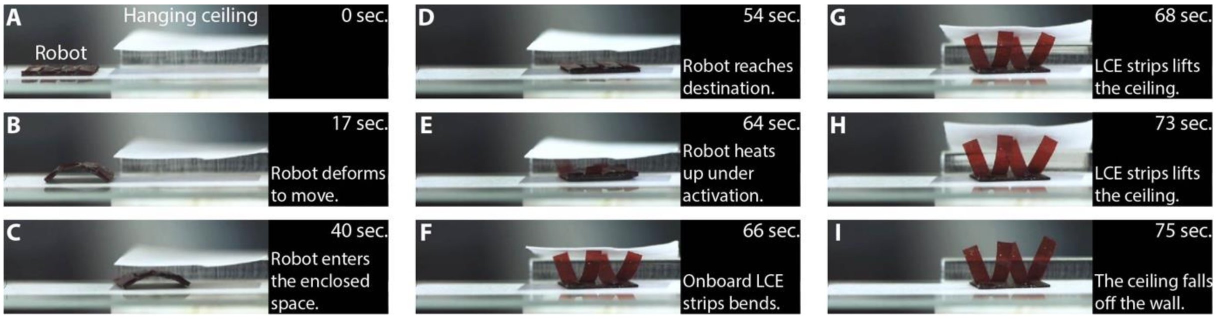

Figure S3. Proof-of-concept demonstration of an exemplar robot activating onboard LCE strips. The robot deformed its body to move like an inchworm from an open space into an enclosed space with a ceiling made of polystyrene. LCE strips was afforded on its back. After reaching its destination, the robot stopped and received energy transferred via the second ETM. The robot heated up and activated the onboard LCE strips, which are heat-sensitive. The LCE strips bent upward and lifted the ceiling until the ceiling was pushed away from the supporting wall. Time-stamped side-view images of this experiment are chronologically displayed from **A)** to **I)**.

Supplementary Videos

Supplementary Video 1. An untethered mobile miniature robot moved within a chamber filled with distilled water preheated to 37 °C with walls made of chicken meat. The robot moved to a target site, performed heat treatment, and moved back to its starting position.

Supplementary Video 2. An untethered mobile miniature robot moved on a flat substrate from an open space into an enclosed space. After reaching the target position, the robot was commanded to activate its onboard laser diode to shine laser light to illuminate the workspace. Afterward, the robot continued to move forward in the enclosed space.

Supplementary Video 3. An untethered mobile miniature robot moved on a flat substrate from an open space into an enclosed space. After reaching the target position, the robot was commanded to activate its onboard LED to shine visible red light to illuminate the workspace. Afterward, the robot continued to move forward in the enclosed space.
